# Response: Commentary: Parent-Reported Behavioral and Psychiatric Problems Mediate the Relationship between Sleep Disordered Breathing and Cognitive Deficits in School-Aged Children

**DOI:** 10.3389/fneur.2018.00063

**Published:** 2018-02-13

**Authors:** Dale L. Smith, David Gozal, Scott J. Hunter, Leila Kheirandish-Gozal

**Affiliations:** ^1^Department of Psychology, Olivet Nazarene University, Bourbonnais, IL, United States; ^2^Department of Pediatrics, Pritzker School of Medicine, Biological Sciences Division, The University of Chicago, Chicago, IL, United States; ^3^Department of Psychiatry and Behavioral Neuroscience, Pritzker School of Medicine, Biological Sciences Division, The University of Chicago, Chicago, IL, United States

**Keywords:** sleep-disordered breathing, cognition, behavior, children, mediation

We appreciate the opportunity to further discuss our recent study on the potential for behavioral and psychiatric functioning to mediate the relationship between sleep-disordered breathing (SDB) and cognitive functioning (1), in the context of the recent commentary raising various critiques on this report (2). In the following paragraphs, it will become clear that such commentary unfortunately falls outside the realm of providing substantive and informative reporting based on an adequate understanding of the current state of sleep medicine as it pertains to SDB in children, neuropsychological assessment, or current practices in statistics.

The bulk of concerns as raised by Barwick and Guilleminault appears to involve our use of several highly validated scales of behavioral and cognitive functioning in children. In support of their arguments, the authors take the somewhat confusing and misleading stance that our discussion of the wide applicability of these measures to various clinical populations does not necessarily imply that such measures are adequate or extensively validated in our population involving sleep-disordered children. A cursory examination of the recent literature in this field should illustrate the common clinical use and relevance of these measures to this child sleep medicine [e.g., review articles such as Ref. (3–5)]. In addition, although concerns regarding use of individual subscores are not particularly novel, the authors gloss over how these measures were used in our report and seem out of touch with how they are currently being extensively applied in this field. We should first state that inferences were never made based exclusively on any of these individual subtests in our report but were instead made regarding general domains, within which these measures have been shown to provide valid information alongside other associated measures. Furthermore, the authors appear to rely on inadequate review of the years of research that has been conducted and subsequently published in peer-reviewed contexts, within the domains of developmental psychopathology, pediatric neuropsychology, adult clinical neuropsychology and psychopathology, and in collaborative research within medicine, where the examination of groups of single test performances as well as aggregate factors from the measures utilized, such as has been done with our studies, are in fact the norm when addressing components of both cognitive and behavioral status. The authors interestingly choose to reference both Wechsler and Sattler [from their citations (2, 3)], ignoring that neither of these individuals ever discussed that research concerning cognitive and behavioral domains of functioning should not be examined at both the individual subtest and aggregate factor level, as we have done with our studies. In fact, what Wechsler and Sattler discuss is the importance of looking at aggregate information when making *clinical decisions*, and not research inferences, something we agree with as well. They also do not reference other informative psychometricians within neuropsychology (i.e., it is important to recognize that neither Sattler nor Wechsler were neuropsychologists), who have addressed how research addressing cognition and behavior can be well informed by the examination of sets of appropriate data [e.g., Ref. (6–10)]. Furthermore, none of the papers referenced by Barwick and Guilleminault expressed any specific rationale as to why both subtest performances and aggregates of subtests contributing to the factors of broader interest could not be useful information and provide informative data regarding cognitive capacities; indeed, they do not address how measures are utilized for research purposes at all. Similarly, while Achenbach’s and Conner’s measures are developed to provide information at a global level, they also have a long standing history of consideration at the individual domain level [e.g., Anxiety; Disruptive Behavior; and Attention (11–14)], particularly with regard to psychopathology and behavioral functioning. The wealth of articles published in premier peer-reviewed journals, including those with some of the highest impact factors in the developmental neuropsychology field (e.g., Child Development; Pediatrics; NEJM; and The Lancet) that includes consideration of subtest and aggregate subscale data is substantial [e.g., Ref. (12, 15–17)], which includes a paper utilizing a single Conner’s hyperactivity subscore as an outcome that was in fact coauthored by Guilleminault (18). That such extensive implementation of such approaches is ignored or glossed-over by Barwick and Guilleminault is unfortunate and misrepresents how researchers in clinical domains frequently and appropriately make use of cognitive and behavioral assessment measures.

Also misleading is the authors’ contention that the plausibility of other model specifications (e.g., cognition as a mediator) was simply not assessed or addressed in our report. Their acknowledged of lack of statistical sophistication could certainly obviate forgiveness for ignoring a cardinal rule for model building; that sound and defensible theory should guide model creation, rather than building such models exclusively on fallible tests of significance or model fit. However, this does not excuse the failure to acknowledge that such alternate model specifications were indeed conducted and were explicitly addressed in our report as footnote #1. These theoretical constructs were explored to ensure defensibility of our proposed model and were found to be inferior at ascertaining mediational effects compared with the *a priori* mediation model that formed the basis for our publication (e.g., non-significant mediational pathways for nearly all iterations of SDB when examining cognition as a mediator).

Barwick and Guilleminault further point to modest relationships between SDB and behavior and cognitive functioning as if the presence of such relationship falling below a specific threshold of strength suggests problems in relevance or explanatory power. This criticism ignores the fundamental notion that a large and highly variable number of factors likely contributes to differences in the context of complex psychological constructs. It is thus unrealistic to expect that the proportion of the variability in behavior or cognition that may be accounted for by clinical factors in any field will be more than simply modest. If as low as 5% of the variability in behavioral or cognitive functioning can be explained by SDB, or could be improved through clinical intervention of related conditions, this would certainly constitute a relevant topic for clinical consideration. Although larger sample sizes can, of course, detect smaller relationships (e.g., a sample of equivalent size to ours could detect correlations as low as *r* = 0.035 with 0.8 power, far smaller than those in our report), *post hoc* naïve regression analyses indicated that SDB status and behavior accounted for greater than 5–10% of the variability in cognition in most iterations of both adjusted and unadjusted models. It is certainly important to be wary of those who may abuse the power inherent to large sample sizes in significance testing; however, expectations that the majority of the variance in cognition, behavior, or psychiatric outcomes will be attributable to sleep pathology suggest poor understanding of this field.

Other analytic concerns that Barwick and Guilleminault raise generally exist for all observational research studies (e.g., omitted variables and potential confounding) and are certainly important to consider in utilizing mediation analyses. However, these were already extensively addressed in our report, making the necessity of reiterating them in a separate commentary questionable. Furthermore, the notion that the possibility of existent unadjusted confounders renders an analytic approach that utilized several existing mediation methods and variable characterizations along with a relevant sensitivity analysis “incomplete” seems out of touch with the realities of virtually all applications of observational research. We appreciate the authors’ attempts to encourage dialog and critical thought regarding this important topic, as this is obviously a vitally important part of the peer review process and the hallmark of progress and scientific understanding. However, when such commentaries contain inaccuracies, are out of touch with current practices or expectations in the field of interest, or simply re-report possible limitations that were already addressed in the original publication, their utility is unfortunately diminished.

## Author Contributions

All authors listed have made a substantial, direct, and intellectual contribution to the work and approved it for publication.

## Conflict of Interest Statement

The authors declare that the research was conducted in the absence of any commercial or financial relationships that could be construed as a potential conflict of interest.
